# Modernising Orodispersible Film Characterisation to Improve Palatability and Acceptability Using a Toolbox of Techniques

**DOI:** 10.3390/pharmaceutics14040732

**Published:** 2022-03-29

**Authors:** Neel Desai, Marc Masen, Philippa Cann, Ben Hanson, Catherine Tuleu, Mine Orlu

**Affiliations:** 1 Research Department of Pharmaceutics, UCL School of Pharmacy, University College London, London WC1N 1AX, UK; c.tuleu@ucl.ac.uk; 2 Tribology Group, Department of Mechanical Engineering, Imperial College London, London SW7 9AG, UK; m.masen@imperial.ac.uk (M.M.); p.cann@imperial.ac.uk (P.C.); 3 UCL Mechanical Engineering, University College London, London WC1E 7JE, UK; b.hanson@ucl.ac.uk

**Keywords:** 3D printing, acceptability, bio-tribology, disintegration testing, mouthfeel, orodispersible films

## Abstract

Orodispersible films (ODFs) have been widely used in paediatric, geriatric and dysphagic patients due to ease of administration and precise and flexible dose adjustments. ODF fabrication has seen significant advancements with the move towards more technologically advanced production methods. The acceptability of ODFs is dependent upon film composition and process of formation, which affects disintegration, taste, texture and mouthfeel. There is currently a lack of testing to accurately assess ODFs for these important acceptability sensory perceptions. This study produced four ODFs formed of polyvinyl alcohol and sodium carboxymethylcellulose using 3D printing. These were assessed using three in vitro methods: Petri dish and oral cavity model (OCM) methods for disintegration and bio-tribology for disintegration and oral perception. Increasing polymer molecular weight (MW) exponentially increased disintegration time in the Petri dish and OCM methods. Higher MW films adhered to the OCM upper palate. Bio-tribology analysis showed that films of higher MW disintegrated quickest and had lower coefficient of friction, perhaps demonstrating good oral perception but also stickiness, with higher viscosity. These techniques, part of a toolbox, may enable formulators to design, test and reformulate ODFs that both disintegrate rapidly and may be better perceived when consumed, improving overall treatment acceptability.

## 1. Introduction

Orodispersible films (ODFs) are single- or multilayer, postage-stamp-sized polymeric thin sheets that rapidly disintegrate in the mouth upon contact with saliva, without the need for additional fluid [1]. ODFs can improve treatment compliance in patients [2] and the films offer a wide range of characteristics including precise dose adjustment, ease of administration and adherence to the oral mucosa [3] making them suitable for addressing the needs of paediatric, geriatric and dysphagic patients [4,5,6,7]. The acceptability of ODFs is defined as “the overall ability and willingness of the patient to use and their caregiver to administer the medicine as intended” [8] and requires careful consideration of both patient and dosage form factors including: swallowability, palatability and administration [9] and has been well studied in key patient demographics [10,11,12,13].

ODF perception, hence overall acceptability, is dependent on the film composition and the film formation process since these determine the physicochemical properties of ODFs which have an impact on the disintegration time, dosage uniformity, drug release and mechanical properties [14].

Water-soluble polymers, such as cellulose [carboxymethylcellulose (CMC), hydroxypropyl methylcellulose (HPMC)], polyvinyl alcohol (PVA), pullulan or starch can be mixed with other excipients including plasticisers, surfactants or taste-masking compounds and/or active pharmaceutical ingredients to produce ODFs [3]. Mussazi et al. describe various ODF fabrication methods, from the simplest procedures utilising solvent casting to more technologically intensive techniques such as electrospinning, hot-melt extrusion and printing, including 3D printing [14]. The physical properties of ODFs, including appearance, structure and thickness, are dependent upon which fabrication process is chosen, although all films must maintain the ability to rapidly disintegrate in the oral cavity [1].

Disintegration time has been observed to be the key characteristic affecting acceptability of ODFs [15]. The European Pharmacopeia necessitates that ODFs must disintegrate in less than three minutes [1]. This guideline, first introduced in 2012, provided a clear discrepancy between dispersible and non-dispersible dosage forms. However, the Pharmacopeial method for calculating ODF disintegration time is not the best with various other methods developed and used in the literature to assess disintegration including: Petri dish [16], drop [16], slide frame and ball methods [17] and oral cavity model (OCM) [18]. These differing methods demonstrate the central role of disintegration both during ODF development and in the ability and willingness of patients to accept the administered oral dosage form.

Taste, texture and mouthfeel of ODFs have also been shown to affect patient acceptability of this dosage form [19]. Batchelor et al. describe medicine mouthfeel as the sensation from the ingestion, mastication (or chewing) and swallowing of the dosage form, all of which are influenced by the physicochemical properties of the administered medicine [20]. The food industry has been the major driver of mouthfeel research in attempts to optimise and maximise pleasure sensations during consumption to establish food choice preferences. Medicines are required out of necessity, and therefore it is not essential that medicines are pleasurable, provided mouthfeel and taste are sufficiently acceptable that they are not a barrier to compliance during administration [20].

Orally administered medicines are subjected to forces at varying speeds and pressures whilst inside the oral cavity [21]. Patients are likely to experience sensory and textural attributes ranging from hard, soft, adhesive and/or sticky, slippery, gritty and creamy dosage forms [22]. During the process of swallowing, there are a range of vertical forces that are distributed across a bolus, which have been modelled and visualised in vitro by the OCM [23]. There are also a range of additional horizontal forces in vivo that must also be considered through appropriate in vitro analysis. Tribological analysis provides an appropriate mechanism to describe and measure horizontal shear against oral dosage forms, therefore elucidating the potential creaminess, slipperiness and adhesion of materials withing the oral cavity.

Tribology is defined as the science of interacting surfaces in relative motion, incorporating the principles of friction, lubrication and wear which are applied in the study of thin film formation between the tongue and upper palate [24,25]. In food science, tribology has been suggested to contribute to the understanding of oral processing, texture and mouthfeel [26] and has demonstrated the capacity as a characterisation tool for correlating with sensory perception [27].

However, traditional tribometers that use steel on both the upper and lower surfaces are unrepresentative of in vivo oral processing, as the desired pressure is much higher between these materials [28]. The design of physiologically relevant tribometers utilises soft and hard surfaces to reduce contact pressure by using softer inorganic material such as polydimethylsiloxane (PDMS), rubber and silicone [29]. Lubrication studies performed using a tribometer should use materials that ideally imitate the mechanical and chemical properties of the human tongue and upper palate [30].

A flat-on-flat tribology experiment setup [31] was adapted such that the two flat surfaces mimicked the tongue-upper palate configuration seen in vivo and in the OCM. The upper surface of the acrylic was loaded and reciprocated against the lower, stationary, silicone tongue surface. The tribology setup was further developed by including simulated salivary fluid (SSF) [32] as the addition of saliva for tribological characterisation has been shown to be of great importance with saliva acting as a lubricant [33], reducing the shear stress on the tongue surface, thus reducing the coefficient of friction [29]. During mechanical processing, the speed between the tongue and palate and temperature were important physical factors that were controlled.

Although the use of tribological assessments for the study of friction and perception of food has been well explored, its use for testing pharmaceutical oral dosage forms is a novel application. Moreover, tribology alone does not provide explicit data on oral processing but when combined with other tools, such as rheology and the OCM, may offer valuable insights into the performance and perception of materials and formulations [20].

The present study aims to highlight that despite advances in ODF fabrication using 3D printing, it is important to determine characteristics that are important to overall medicine acceptability such as disintegration, taste, texture and mouthfeel. This paper describes the use of the Petri dish and OCM methods of disintegration to provide formulators with more relevant testing parameters and feedback on ODF performance. This study also describes the first use of physiologically relevant tribology testing of ODFs to assess disintegration and the potential correlation of lubricating properties to in vivo oral perception. We highlight the use of the described tests as individual tools in a formulator’s toolbox, which could provide knowledge and data to help guide the design of oral formulations.

## 2. Materials and Methodologies

### 2.1. Materials

The four ODF types were prepared using either sodium CMC Blanose^™^ 12M31P (MW = 395 kDa) and Blanose^™^ 7HF-PH (MW = 725 kDa) acquired from Ashland (Wilmington, DE, USA) or PVA: Emprove^®^ 4–88 (MW = 39 kDa) and Emprove^®^ 40–88 (MW = 197 kDa) acquired from Merck-Millipore (Burlington, MA, USA). The films were coloured with 0.1% *w*/*v* Sulforhodamine B (dye content 75%) solution (Merck-Millipore, Burlington, MA, USA) to aid visualisation within the BTM and OCM. The composition and preparation of SSF was according to the formulation reported by Gittings et al. [32].

### 2.2. ODF Preparation and Production

The ODF polymer stock solutions were prepared by weighing and dissolving the required masses of Blanose^™^ and Emprove^®^ in 100 mL of DW mixed with Sulforhodamine B dye (see Table 1). The suspensions were placed on a hotplate stirrer (between 70 and 90 °C), with a magnetic stirrer bar (speed setting 5) for one hour to aid complete polymer dissolution and dispersion of air bubbles. Once dissolved, the polymer stock solutions were allowed to cool to room temperature before being 3D printed.

The ODFs were designed using Onshape (Onshape Inc., Boston, MA, USA), with 30 × 20 mm dimensions. The designs were exported as stereolithography (.stl) files to the Bio X bioprinter (Cellink, Gothenburg, Sweden) for printing, according to the parameters in Table 1. Once printed, ODFs were allowed to dry overnight at ambient conditions, cut to the original 30 × 20 mm dimensions where spreading was observed and transferred to a desiccator for final drying.

### 2.3. ODF Stock Solution Rheology

Rheological profiles of the ODF polymer stock solutions were determined using rotational rheometry with a Bohlin Gemini HR Nano Rheometer (Malvern Panalytical, Malvern, UK). The rheometer was set up with a cone and plate attachment (40 mm diameter, 4° angle), the heat-plate set to 25 °C and the gap height was 150 μm. The four stock solutions were subjected to 30 different shear rates, ascending logarithmically from 0.01 to 100 1/s and the measurements recorded in triplicate (*n* = 3).

### 2.4. BTM Disintegration

**Figure 1:**
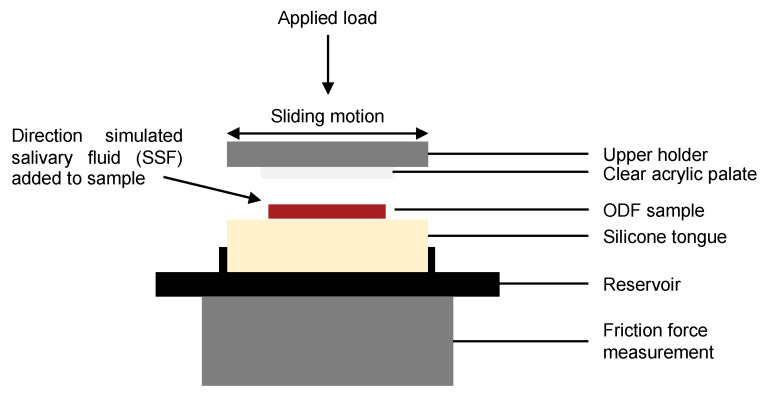
Diagrammatic representation detailing the key components of the BioTribometer setup.

To determine the physiological mechanical stresses applied to the ODFs during disintegration in the oral cavity, a BioTribometer (BTM; PCS Instruments, London, UK) capable of measuring the friction properties of lubricated and unlubricated contacts was used [29]. In the BTM setup, a stationary tongue model made of silicone was inserted into a 3D printed reservoir, designed to capture any tongue surface fluid run-off, and loaded against a flat, circular acrylic sheet representing the hard palate of the oral cavity (see Figure 1) and stored in a temperature-controlled environment.

The tongue, formed of silicone (Dragon Skin™ 10 Medium; Smooth-On Inc., Macungie, PA, USA) was cast such that the final dimensions were 35 × 35 × 7 mm. The top specimen, comprising a circular acrylic sheet (30 mm diameter × 5 mm height) glued to the upper holder, was brought into reciprocating sliding contact with an ODF sample positioned on the silicone tongue surface with a specific load and motion (stroke length and frequency, see Table 2). The transparent top specimen allowed observation of ODF disintegration during BTM testing.

The top specimen was independently actuated in three directions [34]. The lower specimen was heated to 35 °C. The coefficient of friction was measured by two force transducers in the bottom platform, and the applied normal load was measured with a third force transducer connected to the reciprocating clear acrylic palate [29,34,35].

For this disintegration study, the ODF sample was positioned at the centre point of the silicone tongue and coefficient of friction was recorded. SSF was introduced manually: 100 μL every four strokes, such that the final flow rate corresponded to 1.5 mL/min [32]. BTM reciprocation cycles and manual delivery of SSF were repeated until ODF disintegration was observed or three minutes had elapsed, whichever was sooner, and the BTM stopped. For all ODFs, friction data were recorded in triplicate (*n* = 3) at 100 Hz continuously over the reciprocation cycle (using LabVIEW Software, National Instruments, Austin, TX, USA).

### 2.5. OCM Disintegration

The disintegration of ODFs in the OCM has been previously studied [18], but the OCM has since been reprogrammed [36] with the updated methodology briefly detailed as follows. Each ODF sample was positioned at the median point of the silicone tongue and the OCM compression sequence initiated. The cavity was continuously irrigated with SSF at a rate of 1.5 mL/min via a syringe driver [32], resulting in a thin layer of SSF forming across the tongue surface with SSF flowing in the anterior-to-posterior direction [36]. Two-second compression sequences were repeated until ODFs disintegrated or three minutes had passed (note: disintegration was an observed feature during OCM testing). A plan view of the ODF was recorded at 30 images per second (Apple iPhone X, Apple Inc., Cupertino, CA, USA); these images were analysed as a measurement of ODF disintegration within the OCM [36].

The recorded video files were examined using an image analysis procedure, developed and written using MATLAB (MathWorks, Natick, MA, USA), where a single frame was extracted and an edge detection method used to locate the ODF perimeter during disintegration from which the area (in pixels) was derived and plotted against time to display disintegration–time profiles for each ODF tested [36]. Each ODF type was tested in triplicate (*n* = 3).

### 2.6. Petri Dish Disintegration

To determine the single disintegration timepoint for the four ODFs, the rectangular film samples were placed in a 90 mm Petri dish and positioned between the springs of a 37 °C water bath under gentle shaking (70 rpm) [37]. Prewarmed SSF (2.5 mL) was deposited directly on top of each film sample [16]. Film disintegration, defined as the point where structural integrity was lost, that is film breaking, as per operator observation from the viewing area [38], was recorded using a stopwatch. Each ODF type was tested in triplicate (*n* = 3).

### 2.7. Data Analysis

The OCM data were analysed using MATLAB (MathWorks, Natick, MA, USA). All other recorded data were visualised and analysed using Prism 9 (GraphPad Software Inc., San Diego, CA, USA). Statistical analysis of the modified Petri dish disintegration test and mean coefficient of friction of the four ODFs was performed using a one-way analysis of variance (ANOVA) with Tukey’s multiple comparison test.

## 3. Results and Discussion

### 3.1. ODF Formation

ODFs are typically manufactured using a solvent casting process, expertly described by Hoffman et al. [39], with the final step necessitating the cutting of individual films of desired dimensions from a film sheet. There are limitations to solvent casting, including lack of content uniformity, dose diversification and safety of solvents [2] which could be improved by using other manufacturing techniques. The present study was designed based on previous work conducted by this research group that formed CMC and PVA ODFs using solvent casting [15]. The study reported that 1% *w*/*v* CMC films had suitable disintegration times and dosage form acceptability in in vivo human panel studies, and this study now looks to leverage the benefits of 3D printing for ODF production.

Polymer concentrations of CMC (1% *w*/*v*) and PVA (5% *w*/*v*) were selected to provide comparison to ODFs previously produced with the same polymer types and grades using the standard solvent casting method [15]. The 3D printing method was optimised to ensure that films produced were stable and maintained structural integrity after printing and during testing.

With 3D printing technologies, there is the potential for material spreading following extrusion from the syringe nozzle. Although a common occurrence, the printed material should maintain structural integrity; therefore, there should be no appearance of spreading [40,41]. The 1% *w*/*v* CMC and 5% *w*/*v* PVA formulations did not prematurely leak from the nozzle and were extruded on command using the nominated parameters in Table 1. All four polymer stock solutions formed reproducible ODFs with good overall resolution and dimensionality following extrusion from the syringe nozzle, part of the printing process. All but the C2 films resulted in minimal degree of spreading, with structural integrity maintained for the majority of films; just 5 of the 35 films prepared required trimming down to the original size.

**Figure 2:**
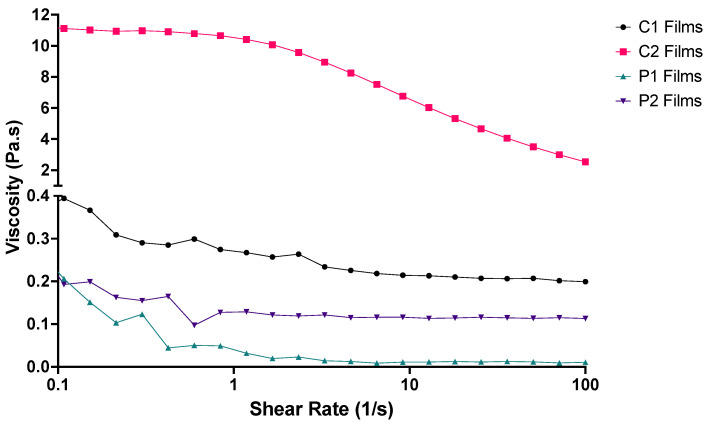
Rheological profiles of the four ODF polymer solutions used for 3D printing the films (*n* = 3).

Polymer solubility is molecular-weight-dependent and has been well established in the literature [42]. The lower solubility of longer chain macromolecules compared to shorter ones of an analogous structure has also been well documented [43]. Rheological analysis of the four polymer stock solutions revealed that increasing polymer MW increased solution viscosity [44]; the comparative viscosity was more pronounced between C1-C2 than P1-P2 stock solutions (Figure 2). This likely a result of lower MW polymers, such as PVA, forming beads rather than fibres in solution and higher MW polymers, such as CMC, forming fibres of larger diameters [45].

At lower shearing forces, C2 was observed to have greater resistance to spreading than the other three polymer solutions (Figure 2). Both C1 and C2 stock solutions demonstrated shear thinning behaviour: when shear rate increased, viscosity decreased, consistent with previous study findings [40,46]. P1 and P2 stock solutions did display some shear thinning characteristics, but plateaus were reached at much lower shear rates as these solutions contained PVA. The shear thinning behaviour demonstrated by C2 eased extrusion through the syringe needle with decreased viscosity at higher shear rates, and ODF formation benefitted from the higher viscosity at lower shear rates allowing the structure to maintain the desired shape.

A previous study by Ong, Steele and Duizer noted that the pharyngeal shear rates during swallowing likely extend above 50 s^−1^, and samples having similar viscosities around this shear rate had perceived viscosities which were not related to rheological measurements alone [47]. This observation may also be true for P1, P2 and C1, whose viscosity at 50 s^−1^ was observed to be similar, but performances in the disintegration tests differed (below). Three-dimensional printing resulted in films of slightly greater thickness than those previously prepared using solvent casting (Table 3).

Traditional film and solvent casting methods require dissolution of active pharmaceutical ingredients and excipients in solvent, casting ODFs using the preferred method, drying, cutting of films into the desired size and shape and packaging ready for patient use [41]. Although both casting methods are simple to execute, drying, temperature and humidity must be carefully controlled as these parameters affect the final film’s properties, including thickness and result in a lengthy process [48,49]. Additionally, solvent casting uses heat to evaporate water from polymer solutions during the initial stages of film formation. ODFs that are dried too quickly using increased temperatures can become brittle and break on handling, whilst using reduced temperatures causes ODFs to not form fully as the formulation spreads and films become impossible to handle. Three-dimensional printing uses no additional heat source. The Bio X bioprinter used in this study was maintained at 15 °C during printing. Natural air-drying of the 3D printed ODFs resulted in greater water retention after the initial overnight drying period and longer drying times overall. The drying process and time limitations affect other ODF fabrication processes, including film and solvent casting, but 3D printing may provide the answer to ensuring homogeneous ODF formation post drying.

### 3.2. ODF Disintegration Studies

ODFs are characterised by their fast disintegration times and are expected to disintegrate within 180 s when placed on the tongue within the oral cavity, as per the European Pharmacopeia [1]. However, there remains a lack of information regarding which methodologies should be applied to determine ODF disintegration time [3]. There is a general consensus that disintegration occurs when structural integrity is lost, resulting in an observed portion of the film being removed from the main ODF structure. Therefore, disintegration can be defined as the time at which an ODF splits from one single structure into two distinct objects. To determine the single ODF disintegration time, a modified Petri dish method was followed. As reported previously by Desai et al., a single time measurement does not provide a complete overview of disintegration, and monitoring changes in disintegration mechanism profiles is likely to provide greater insight [36]. This therefore prompted assessment of the printed ODFs in the OCM and BTM.

The in vitro disintegration time measured by the modified Petri dish methodology resulted in relatively fast disintegration of P1, P2 and C1, all within the first 60 s of testing (Table 4). C2 demonstrated a much longer disintegration time, greater than three minutes in all cases. A statistically significant difference was found between all four ODF samples (*p* < 0.0001). Observed ODF disintegration was due to the dissolved polymers in each stock formulation; the differences between C1–C2 and P1–P2 were a direct result of polymer MW, since the comparative films had equal composition and dimensions.

The disintegration behaviour and mechanism of polymeric ODFs is closely related to MW of film-forming polymers and intermolecular bonding [50]. Films comprising smaller polymers dissolve quicker than those that contain larger polymers [39,51,52]. Polymers of lower MW, such as the PVA used in this study, have a lower degree of molecular interaction because the polymer chains are shorter, allowing SSF to access the films more and enable rapid disentanglement of polymer molecules in solution [15,53]. ODF disintegration kinetics were therefore dependent on polymer MW and chain structure.

**Figure 3:**
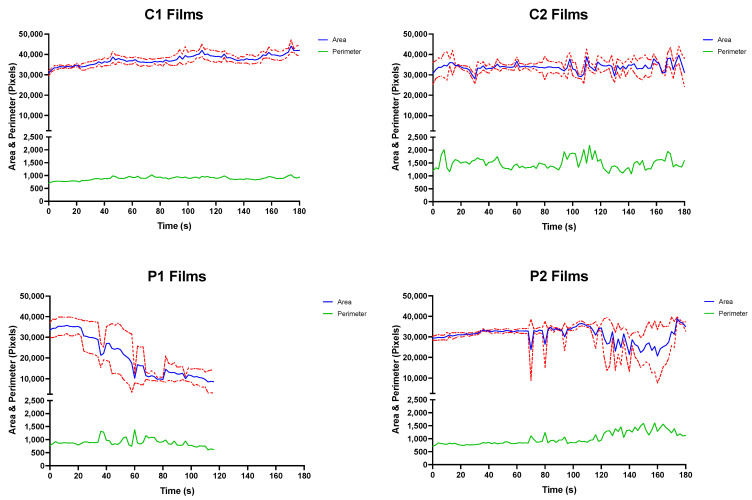
Mean top-down area-time and perimeter–time profiles for C1 (**top left**), C2 (**top right**), P1 (**bottom left**) and P2 (**top right**) ODFs assessed in the OCM with standard deviations (*n* = 3).

The OCM was used to assess the mechanism of film disintegration expected to occur in vivo over time. Here, the recorded disintegration of all films was substantially longer when compared to the same ODFs assessed using the modified Petri dish method (Table 4). In fact, only the P1 ODFs were observed to have achieved disintegration inside the OCM during the experimental process. This was evident from the observed and measured performance of the P1 ODFs in the OCM during the simulation (Figure 3; bottom left).

Closer examination of the P1 disintegration–time profile revealed a slight decline in remaining area at approximately 24 s, coinciding with the removal of film fragment of the tested ODFs from the silicone tongue surface.

The larger P1 ODF remnant continued to disintegrate inside the OCM with each compression sequence, with smaller disintegrate product continuing to be removed from the tongue surface. The presence of the shrinking larger ODF fragment on the silicone tongue surface provides explanation for the sufficient film area remaining even after the measured endpoint. The remnants of P1 films were eventually “washed away” by SSF, and testing stopped at 120 s.

Observations made during the swallowing sequences with P2 ODFs showed that some tearing of the films did occur at 70, 80 and 95 s (Figure 3; bottom right). However, these smaller fragments, all occurring at the bottom right corner of the ODFs, later coalesced with the main film structure. Similar observations were made between 105 and 160 s where a larger tear formed down the vertical length of the film, resulting in the image analysis procedure reporting a reduced ODF area. As was seen at earlier timepoints, the ODF area returned to the approximate starting value as the P2 films reassembled.

P2 films may have reformed because of PVA MW used in the stock formulation. PVA structures reportedly have shape memory properties with PVA polymer chains readily forming inter-chain hydrogen bonds [54]. Formulating the P2 stock solution may have resulted in melting of polymer crystals, breaking some hydrogen bonds. The ambient temperatures of storage and cooler conditions during printing may have allowed the formation of new “temporary” non-covalent or dynamic covalent cross-links intertwined between the “permanent” covalent or non-covalent hydrogen cross-links between polymer chains that did not melt during stock solution formation [55].

Zero disintegration was observed or measured when C1 and C2 (Figure 3; top left and top right) ODFs were assessed in the OCM. Spreading across the acrylic palate of C1 and C2 films was noted as testing duration and number of compressions performed increased, which caused thinning at ODF corners. Unlike the PVA ODFs, the CMC films were observed to stick to the OCM upper acrylic palate when inspected side-on to the apparatus. Here, the ODFs partially swelled, resulting in the films becoming wider and eroding from the underside through co-action of a thin film of SSF and compression exerted onto the dosage form by the OCM artificial silicone tongue—similar to a previous OCM study examining orodispersible tablets [36]. After 180 s, the C1 and C2 ODF structures appeared to remain intact from the plan view, although a thin layer was visually observed to have “washed away” from all the tested films by the SSF flowing down the tongue surface in the anterior-to-posterior direction. The thickness of remaining ODFs was not recorded.

There is likely a polymer MW limit beyond which the reasonably low volumes of SSF used in the OCM causes little to no ODF disintegration. This limit is likely close to 200 kDa since the P2 films (MW = 197 kDa) were observed to significantly tear during OCM assessments, but self-formed due to the inherent properties of PVA described above.

The four sequential stages of swallowing have been well documented and described since the first mammalian studies were conducted [56]. The first two stages are represented in the OCM, briefly summarised as follows. A bolus is held in the anterior section of the tongue surface against the hard palate during the oral preparatory phase [57]. The anterior tongue then rises to contact the hard palate whilst the posterior tongue lowers and the whole tongue surface moves upwards increasing the contact with the palate from anterior to posterior, squeezing and propelling the bolus posteriorly along the palate and into the pharynx during the oral propulsive stage [57].

OCM assessment of all C1 and C2 ODFs found that the films adhered to the upper acrylic palate and remained attached for the duration of testing. To reflect the horizontal movement of a bolus across tongue tissue, from anterior to posterior, in contact with the hard palate as described above, tribology testing was performed on all four ODFs using the BTM. The testing procedure involved use of a reciprocating flat-on-flat configuration, simulating the conditions and motions of in vivo tongue–palate contact [29]. In addition to OCM testing, BTM tribological analysis allowed for more physiologically relevant estimations of in vivo disintegration by exposing only a single large surface of the ODF to salivary fluid in vitro, as seen in the human oral cavity.

Overall, ODF disintegration of all film types occurred much faster under shear loading with the BTM than with the modified Petri dish or OCM methods; all films were observed to have disintegrated within approximately 30 s of starting testing (Table 4). In contrast to the two aforementioned methods, disintegration was quickest with the highest MW polymer formulation (C2) and slowest with the formulation comprising the lowest MW polymer (P1).

Visual observations of ODF disintegration were noted by the BTM operator. P1 and P2 films were seen to tear from the outer edges causing ODFs to break into smaller fragments. In contrast, C1 and C2 films were seen to disintegrate from the centre outwards with ODFs spreading before separation. Unlike OCM testing, the ODFs did not adhere to the reciprocating acrylic palate attached to the upper holder. The disintegration times recorded using the BTM demonstrate the influence of shear forces applied to ODFs when placed between the upper acrylic palate and stationary silicone tongue.

In addition to the observed disintegration, the tribological test apparatus calculated the coefficient of friction—the ratio of the force of friction between two bodies and the force pressing them together [58]. Materials with smaller coefficients of friction are considered to be more lubricous [59]. In this study, the coefficient of friction was determined between the acrylic palate and silicone tongue with an ODF sample and SSF placed in between and measured by a force meter. By establishing the coefficient of friction, BTM testing may provide insights into oral sensory perception. This could be important since all ODFs had remnants present on the OCM silicone tongue, even when disintegration was observed (P1), which may be aversive to some individuals.

**Figure 4:**
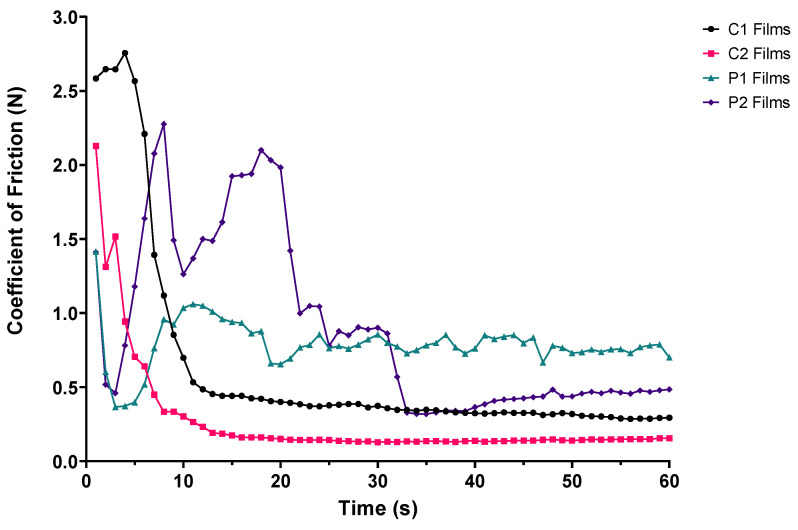
Coefficient of friction–time plot of ODF samples positioned between the upper acrylic palate and silicone tongue during BTM tribological testing (*n* = 3).

When coefficient of friction data were plotted against time (see Figure 4), two key findings were noted. First, the films composed of CMC (C1 and C2) demonstrated a rapid decline in coefficient of friction immediately prior to disintegrating and stabilisation of friction values. Secondly, the films composed of PVA (P1 and P2) started at similar coefficients of friction, followed by a phase of friction fluctuations (decrease–increase–decrease) before ODFs disintegrated, after which friction values plateaued. The differences seen could be explained by the processes observed during ODF disintegration. Tearing of P1 and P2 films from the outer edges inwards increased exposure of the reciprocating upper palate to the silicone tongue, reducing the measured coefficient of friction, whilst film formation resulted in the opposite. This frictional variation was more pronounced with ODFs formed with P2 than P1, given the reassembly observed by P2 films during OCM and BTM disintegration testing.

**Figure 5:**
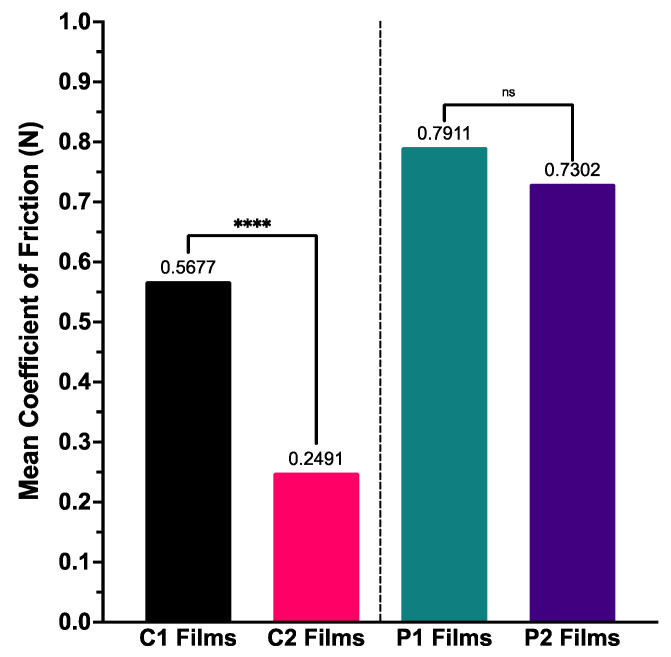
Mean coefficient of friction calculated for ODF samples for BTM tribological testing (*n* = 3), with one-way ANOVA statistical significance data displayed—**** (*p* < 0.0001) and ns (not significant).

Calculation of mean coefficient of friction over the duration of experimentation demonstrated trends similar to those seen with BTM disintegration (Figure 5); coefficient of friction was smallest with the highest MW formulation (C2) and largest with the formulation containing the lowest MW polymer (P1). The differences between C1–C2 coefficients of friction were statistically significant (*p* < 0.0001), but those amongst P1–P2 were not.

This paper presents the first physiologically relevant tribology testing of solid oral dosage forms, after the introduction of tribological studies into pharmaceutical development by Batchelor et al. on liquid oral dosage formulations [20]. The findings from the friction data obtained through tribological analysis have two potential explanations, which may be independent of each other.

Firstly, regarding viscosity of polymer stock solution, the findings from this study state that films formed of higher MW polymer (C2), have higher viscosity when dissolved and once formed into film structures have a lower coefficient of friction between surfaces representative of the oral cavity. ODFs of lowest MW (P1) showed the opposite. This viscosity–coefficient of friction relationship has been documented in the literature across tribology fields [29,60,61]. Secondly, for ODF thickness, our findings showed films comprising lower polymer MW (P1) produced thinner film structures with the fastest disintegration times and highest coefficients of friction. The disintegration performance of these thinner films may be a result of lubrication provided by SSF, altering film surface texture causing an increase in SSF flow and thus increased shear across the ODF surface, increasing friction whilst reducing disintegration time [62].

Previous work by the research group explored manually casted ODFs of the same composition in human sensory panels [15]. The study found that participants found higher MW films (C1 and C2) uncomfortable due to the perceived stickiness of the samples, where the “gummy nature” of films affected mouthfeel and palatability [19], and healthy volunteers preferred the rapidly disintegrating PVA ODFs. The in vitro Petri dish and OCM findings from this study appear to match disintegration time data previously recorded in a human panel, whilst the BTM data appear to corroborate the in vivo perceptions and performance of ODFs.

The paper reports on the use of one possible alternative method to traditional film and solvent casting, 3D printing. The advantages of printing ODFs include greater control over the printing process by setting the desired dimensions and shape, faster film formation and reduced wastage. The primary limitations of 3D printing ODFs include time for optimising the printing process and drying time once produced [41]. However, these limitations also apply to traditional film-forming techniques.

Whilst there have been significant advancements in the production of ODFs, more physiologically relevant in vitro quality control assessment has not seen the same level of progression. This study has reported on three in vitro tools to analyse ODFs, forming part of a larger toolbox which could be used to assess palatability and perception of oral dosage formulations. The modified Petri dish method allows operators to quickly assess ODFs using a minimised setup to determine disintegration time. The OCM provides a unique opportunity to ascertain disintegration behaviour profiles by assessing changes in the observable area during oral processing whilst visualising potential ODF sticking to the upper palate. Through studying disintegration and friction of mechanically degraded ODFs, tribological analysis with the BTM enables the horizontal shear forces applied to oral dosage forms when rubbed between the tongue and upper palate to be replicated whilst providing visual observations to establish reasons why ODFs may adhere to the upper palate during OCM testing. The configurations of all three tests mean that a single ODF is subjected to repeated shear at only a single large surface, representative of in vivo disintegration mechanics with physiologically relevant fluid volume and flow. At present, the dataset explored, whilst broad, was limited in number; hence, we do not have sufficient data to propose a model for the complex relationship of disintegration, friction and in vivo sensory perception of ODFs. However, the results from this study have shown that polymer MW is the most influential factor when producing single component ODFs. Molecular weight affects the rheological properties of stock solutions, which in turn alters the thickness of films formed and observed disintegration time and frictional force.

## 4. Conclusions

Advancements in ODF fabrication technologies, particularly 3D printing demonstrated here, have enabled films to be formed with good resolution, structure and dimensionality. Rheological examination of higher MW polymer stock formulations exhibited their higher viscosity than lower MW polymer suspensions, as expected. Three differing disintegration approaches were examined. The Petri dish and OCM methods demonstrated an exponential relationship between polymer MW and disintegration time, although not all ODFs achieved disintegration inside the nominal 180 s defined by the European Pharmacopeia. Moreover, films of higher MW were observed to adhered to the upper palate inside the OCM, an observation seen previously in vivo. ODF analysis using physiologically relevant tribology apparatus demonstrated that higher MW polymer films disintegrated fastest, and these films had the smallest coefficient of friction. The BTM findings may suggest good oral palatability and mouthfeel of ODFs comprising higher MW polymers, an observation that was noted in previous in vivo studies. These individual tools, when used together as a toolbox of techniques, could allow formulators to have quality control measures that are in line with advancements in ODF formulation and empower formulators to design, test and reformulate ODFs that not only disintegrate rapidly but may also be better perceived when consumed, improving overall treatment acceptability.

## Figures and Tables

**Table 1:** ODF polymer formulation solution composition and optimised 3D printing parameters.

ODF ID	ODF Polymer Stock Solution	3D Printing Parameters					
		Needle Gauge (Diameter)	Compressed Air Pressure (kPa)	Printing Speed (mm/s)	Infill Pattern	Infill Density	Print Cycle Progression
C1	1% *w*/*v* Blanose ™ 12M31P, 0.1% *v*/*v* dye	22 G (0.41 mm)	50	20	Grid infill	12%	30%
C2	1% *w*/*v* Blanose ™ 7HF-PH, 0.1% *v*/*v* dye						
P1	5% Emprove^®^ 4–88, 0.1% *v*/*v* dye		15			25%	20%
P2	5% Emprove^®^ 40–88, 0.1% *v*/*v* dye		70				40%

**Table 2:** Test parameters used for the BTM to mimic physiological oral cavity conditions [29,34].

Parameter	BTM Test Setting
Reciprocating frequency	1 Hz
Applied load	1 N
Approximate contact pressure	≈30 kPa
Sliding velocity	30 mm/s
Stroke length	7.5 mm

**Table 3:** Thickness of ODFs produced by 3D printing and solvent casting methodologies with standard deviations (*n* = 3).

ODF ID	3D Printed Thickness (μm)	Solvent Casting Thickness (μm) ^†^
C1	73.63 ± 2.12	68.33 ± 2.11
C2	79.54 ± 1.96	62.67 ± 2.84
P1	35.25 ± 1.39	24.00 ± 1.31
P2	38.78 ± 1.25	22.00 ± 1.36

^†^ Thickness values derived from previous work by Scarpa et al. 2018 [15].

**Table 4:** ODF mean disintegration times using the modified Petri dish, OCM and BTM methodologies, with standard deviations (*n* = 3).

ODF ID	Petri Dish Disintegration (s)	OCM Disintegration (s) *	BTM Disintegration (s) ^†^
C1	25.3 ± 0.7	>180.0	11.0 ± 2.0
C2	>180.0	>180.0	8.0 ± 2.0
P1	7.4 ± 0.6	24.0 ± 2.0	31.0 ± 3.0
P2	22.2 ± 0.9	>180.0	21.0 ± 2.0

* Observed ODF disintegration by OCM operator, based on the number of compression sequences completed, where one compression cycle corresponds to two seconds. ^†^ Observed ODF disintegration by BTM operator, based on the number of rubbing/shearing cycles completed, where one cycle corresponds to one second.

## Data Availability

The data presented in this study are available upon request from the corresponding author.
